# Pro-viral DNA and antiretroviral treatment simplification

**DOI:** 10.1186/1758-2652-13-S4-P143

**Published:** 2010-11-08

**Authors:** D Zucman, C Rouzioux, V Avettand-Fenoel

**Affiliations:** 1 Foch Hospital, Internal Medicine, Suresnes, France; 2 Université Paris-Descartes, CHU Necker-Enfants Malades, Virology Unit, Paris, France

## 

The question of Highly Active Antiretroviral Treatment (HAART) simplification is important due to long term toxicity and cost of HAART.

Pro-viral HIV DNA associated with Peripheral Blood Mononuclear Cells (PBMC) is a marker of the HIV reservoir which is predictive of the evolution of HIV infection. It remains the only virological quantitative marker detectable during suppressive HAART. A pro-viral DNA > 2.7log before treatment interruption is predictive of a decline of CD4 T lymphocytes after interruption.

We measured PBMC's pro-viral DNA in patients treated with triple nucleoside analogue therapy containing zidovudine who had developed severe lipodystrophy and/or metabolic abnormalities. A switch to a nucleoside analogue bitherapy by stopping zidovudine was proposed for patients whose pro-viral DNA was < 2.7 log. We selected 11 patients (mean age 48 years, 5 women, mean duration of infection=10 years, mean CD4 nadir=288/mm^3^, mean plasma HIV RNA zenith =4,44 log). Patients were on triple nucleoside analogue therapy for 7 years with plasma HIV RNA< 40 copies/ml ; mean CD4 cells = 600/mm^3^. Mean pro-viral DNA was 2,43 log copies/million PBMC. Treatment was switched to abacavir+lamivudine in 5 patients and emtricitabine+tenofovir in 6.

After switch, with a mean follow up of 40 months, plasma HIV RNA remained< 40 copies/ml without blip and CD4 cells remained stable.

In conclusion, treatment simplification with an analogue nucleoside bitherapy is possible in a selected group of patients who were treated with a triple nucleoside analogue therapy in virological success and had a PBMC’s associated pro-viral DNA < 2.7 log before treatment simplification.

Avettand-Fenoel V, Boufassa F, Galimand J, Meyer L, Rouzioux C (2008) HIV-1 DNA for the measurement of the HIV reservoir is predictive of disease progression in seroconverters whatever the mode of result expression is. J Clin Virol 42: 399-404

